# Family reaction to coming out (FRCO): A Spanish adaptation and validation of perceived parental reactions scale

**DOI:** 10.1111/famp.13047

**Published:** 2024-08-08

**Authors:** Juan E. Nebot‐Garcia, Rafael Ballester‐Arnal, Estefanía Ruiz‐Palomino, Olga Fernández‐García, María Dolores Gil‐Llario

**Affiliations:** ^1^ Department of Basic and Clinical Psychology, and Psychobiology Universitat Jaume I Castellón de la Plana Spain; ^2^ Department of Developmental and Educational Psychology Universitat de València Valencia Spain

**Keywords:** disclosure, LGBT, reaction to coming out, relatives, sexual minority

## Abstract

Family reactions to coming out can affect the mental health of individuals who disclose their sexual orientation or gender identity. Therefore, it is important to have an appropriate tool to assess them. The Perceived Parental Reactions Scale (PPRS) assessed perceived parental reactions to the disclosure of gay, lesbian, or bisexual sexual orientation by their children. We adapted the PPRS so that it can be answered by any individual belonging to a sexual or gender minority, and can be answered regarding any member of the family, not just parents. A total of 2627 individuals from Spain participated in this study, with a mean age of 31.59 (*SD* = 11.26). Participants completed the adapted PPRS questionnaire, now named the Family Reaction to Coming Out (FRCO). The FRCO assessed family reactions when disclosing their sexual orientation or gender identity. The majority of participants identified as cisgender men (47.5%) or cisgender women (44.9%), and as gay/lesbian (51.9%). A one‐factor model emerged through exploratory factor analysis (EFA) and confirmatory factor analysis (CFA). The FRCO displayed excellent internal consistency and demonstrated good levels of invariance for participants' gender (male vs female vs nonbinary gender), family member's gender (male vs female), and type of family member (parents vs other family member). Supporting convergent validity, the FRCO has shown a positive correlation with fear of family reaction to coming out. These findings support the validity and reliability of the FRCO tool in assessing the reactions of any family member within the Spanish context.

“Cisheteronormativity” is a set of assumptions that maintain that only two genders exist, that these two genders are determined by biological sex, and that only sexual attraction between these two “opposite” sexes is natural and acceptable (Habarth, 2008; Herz & Johansson, 2015; Schilt & Westbrook, 2009). Therefore, when a person is developing their identity and realizes that they deviate from this norm, they often feel compelled to disclose their sexual orientation or gender identity to their closest circle, such as their own family. In addition to the stress that this process of disclosure can cause (Matthews & Salazar, 2012), individuals belonging to a sexual or gender minority also have to face possible negative reactions from their family members.

Depending on the family's reaction, the person coming out of the closet will experience varying degrees of emotional impact. In a study conducted in the United States, Puckett et al. (2015) found that lesbian, gay, and bisexual (LGB) adults who had perceived greater parental rejection at the time of sexual orientation disclosure exhibited higher psychological distress in the present. In Canada, D'amico et al. (2015) analyzed parental reactions to their LGB children coming out and observed that higher levels of parental support were associated with lower suicidal ideation among the youth. Conversely, the more parents tried to change their children's sexual orientation, the more psychological distress young people experienced. Lastly, in another study carried out in the United States with adolescents and young individuals identifying as lesbian, gay, bisexual, transgender, or questioning, it was observed that those who, during their identity disclosure, received more negative reactions from their families (parents and siblings) exhibited higher levels of depression and lower self‐esteem at the moment of the study (McCurdy et al., 2023).

Given that perceived family's reaction to coming out of the closet appears to be a highly relevant variable for the mental health of lesbian, gay, bisexual, trans, intersex, and other sexual and gender minorities (LGBTI+), it becomes important to have appropriate instruments for its evaluation. In this regard, some studies used a question Likert‐type to assess the family reaction to sexual orientation disclosure (D'Augelli et al., 2008; McCurdy et al., 2023) or a general interview (D'amico et al., 2015), but the vast majority (Baiocco et al., 2012, 2015, 2016; Bregman et al., 2013; Gertler, 2014; Li & Samp, 2021; Michli & Jamil, 2022; Mitrani et al., 2017; Puckett et al., 2015; Richter et al., 2017; Walker, 2016; Wigderson et al., 2019) used the Perceived Parental Reactions Scale (PPRS, Willoughby et al., 2006, 2010), a 32‐item questionnaire that assesses the perception of the initial reaction of the father or mother to the coming out of their LGB children. Even though this scale has been widely used, neither the original articles nor the studies using this scale conducted an exploratory or confirmatory factor analysis.

Although the vast majority of previously mentioned studies focused on family reactions to coming out, some authors have also observed that the reactions of other significant individuals, even if they were not part of the immediate family, also had an emotional impact on the person coming out. In a study conducted on LGB adults residing in the United States, United Kingdom, and Canada, it was observed that negative reactions from close social networks to coming out had long‐lasting effects on the individual's well‐being. However, positive reactions did not show any effect. More specifically, it was found that experiencing a negative reaction from any significant person (the first person they told, the father, the mother, or the best friend) was associated with higher levels of depression and lower self‐esteem, though this effect was significant for self‐esteem only in the case of reactions from the father or the best friend (Ryan et al., 2015).

Therefore, it is necessary to have measures to assess the reaction to coming out, not only from parents but also from any family member. For instance, Li and Samp (2021) adapted the PPRS to evaluate the reaction of heterosexual partners to coming out. Furthermore, the Perceived Parental Reactions Scale (Willoughby et al., 2006, 2010) focuses only on LGB individuals despite the fact that there are other sexual minorities who do not identify with these orientations or that are not cisgender, such as pansexual, asexual, trans, or nonbinary individuals.

## This study: Spanish context

It may seem that in contexts with great social acceptance of the LGBTI+ people, it is not so necessary to study family reactions to coming out, but in Spain, despite a significant majority of people (89%) holding a positive attitude toward gay and lesbian individuals (Pew Research Center, 2020), 8% of Spanish LGBT+ people (aged 15 years and over) reported experiencing physical and/or sexual attacks in the last 5 years due to their sexual orientation, and 41% had experienced harassment in the preceding 12 months because of their sexual orientation (European Union Agency for Fundamental Rights, 2020). Furthermore, as a result of Spain's historical past and Franco's dictatorship, there exists a segment of society that is highly conservative. A clear example of this is the presence of political parties strongly opposing the rights of the LGBTI+ community in the Parliament (Abou‐Chadi & Finnigan, 2019; Rama et al., 2021; Rodríguez‐Temiño & Almansa‐Sánchez, 2021). For this reason, it seems relevant to study the family reaction to coming out in Spain using an instrument adapted and validated for our sociocultural context.

We adapted the PPRS to Spanish context and so that it could be answered by any individual belonging to a sexual or gender minority, and they could respond while thinking about any family member. Additionally, we aimed to conduct the necessary analyzes to examine the scale's factorial structure, internal consistency, convergent validity, and its invariance based on the type of family member (parents vs other family member). Furthermore, given that gender has proven to be a differential variable in attitudes toward the LGBTI+ population (Holland et al., 2013; Worthen, 2012), we also analyzed the invariance based on the participant's gender and the chosen family member's gender.

## METHOD

### Participants

A total of 2627 individuals residing in Spain participated in this study. The average age of the sample was 31.59 years (*SD* = 11.26), ranging from 18 to 75 years. As shown in Table 1, the vast majority were cisgender men (47.5%) or cisgender women (44.9%), identified as gay/lesbian (51.9%), of Spanish nationality (93.4%), atheist or agnostic (75.9%), with a progressive political ideology (76%), and had university education (60.8%).

**TABLE 1:** Main sociodemographic characteristics of participants.

	Total (*n* = 8953)		EFA (*n* = 4416)		CFA (*n* = 4537)		*t*	*df*	*p*	Cohen's *d*
	*M* (SD)		*M* (SD)		*M* (SD)					
Age	31.59 (11.26)		31.60 (11.44)		31.57 (11.07)		0.06	2625	0.948	0.002
	*n*	%	*n*	%	*n*	%	*χ* ^*2*^	*df*	*p*	*Cramer's V*
Gender identity										
Cis men	1247	47.5	638	47.8	609	47.1	1.35	4	0.853	0.023
Cis women	1180	44.9	592	44.4	588	45.4				
Trans men	46	1.8	21	1.6	25	1.9				
Trans women	34	1.3	18	1.3	16	1.2				
Nonbinary gender	120	4.6	65	4.9	55	4.3				
Sexual orientation										
Heterosexual	24	0.9	13	1	11	0.9	2.22	6	0.898	0.029
Gay/Lesbian	1363	51.9	697	52.2	666	51.5				
Bisexual	988	37.6	490	36.7	498	38.5				
Pansexual	176	6.7	96	7.2	80	6.2				
Asexual	58	2.2	28	2.1	30	2.3				
Demisexual	15	0.6	8	0.6	7	0.5				
Other	3	0.1	2	0.1	1	0.1				
Nationality										
Spanish	2454	93.4	1238	92.8	1216	94	1.68	2	0.433	0.025
Spanish co‐nationality	15	0.6	8	0.6	7	0.5				
Foreigner	158	6	88	6.6	70	5.4				
Religious beliefs										
Practicing believer	109	4.1	53	4	56	4.3	0.25	2	0.884	0.010
Nonpracticing believer	525	20	269	20.2	256	19.8				
Atheist or agnostic	1993	75.9	1012	75.9	981	75.9				
Political ideology										
Conservative	73	2.8	38	2.8	35	2.7	1.93	3	0.586	0.027
Center	306	11.6	148	11.1	158	12.2				
Progressive	1997	76	1012	75.9	985	76.2				
Indifferent	251	9.6	136	10.2	115	8.9				
Level of education										
Without studies	2	0.1	1	0.1	1	0.1	6.57	5	0.255	0.050
Primary	27	1	13	1	14	1.1				
Secondary	427	16.3	223	16.7	204	15.8				
Vocational training	572	21.8	268	20.1	304	23.5				
Diploma/Bachelor/Degree	1020	38.8	542	40.6	478	37				
Master/Doctorate	579	22	287	21.5	292	22.6				

### Instruments

In addition to sociodemographic data, sexual orientation, and gender identity, all participants answered questions about their coming out experience with their family members. Firstly, they were asked if they had come out with someone, if they had revealed it to them themselves, and if there was anyone in their family who knew their LGBTI+ identity. Those who answered “yes” to the three questions, indicated the most relevant relative with whom they had come out of the closet. The options to choose from were as follows: “father,” “mother,” “brother,” “sister,” “uncle,” “aunt,” “male cousin,” “female cousin,” “grandfather,” “grandmother,” “partner,” “children,” or “other.” Regarding that family member, they were asked to respond to their reaction to their coming out using the Family Reaction to Coming Out (FRCO) questionnaire, which is the Spanish adaptation we made of the PPRS (Willoughby et al., 2006, 2010).

The original PPRS scale used a unifactorial model to evaluate eight theoretical dimensions of perceived parental reactions, including negative shock (items 13, 18, 23, and 28), denial (items 14, 19, 24, and 29), anger (items 15, 20, 25, and 30), bargaining (items 16, 21, 26, and 31), depression (items 17, 22, 27, and 32), acceptance (items 1, 5, 8, and 10), general homophobia (items 3, 6, 9, and 11), and parent‐focused concerns (items 2, 4, 7, and 12). The internal consistency demonstrated by this scale was excellent (*α* = 0.97), along with good test–retest reliability (*r* ≥ 0.95) and adequate construct validity (Willoughby et al., 2006, 2010).

In the Appendix S1, the English and Spanish version of our adapted scale can be seen. This adaptation consists of 28 Likert‐type items ranging from 1 (*strongly disagree*) to 5 (*strongly agree*), and includes items such as “cried tears of sadness,” “told me it was just a phase,” or “supported me (reverse‐coded item).” The total score ranged from 28 to 140, with higher scores indicating a more negative reaction from the family member.

In addition, participants answered an ad hoc question about what aspects prevented (or had prevented) them from revealing their sexual orientation to their loved ones. There were 12 possible causes, and they had to indicate for each one whether it applied or not. For this study, we selected three options related to the reaction of others: “fear of them getting angry,” “fear of being thrown out of the house,” and “fear of being punished in some way.”

### Procedure

Following the committee approach to translating questionnaires (Maneesriwongul & Dixon, 2004), two psychology experts, specializing in the field of sexual and gender diversity and with a good level of English, translated the PPRS (Willoughby et al., 2006, 2010) into Spanish. Both experts independently translated it and later shared their translations. A third expert reviewed the translation and compared it to the original. The three individuals made the necessary modifications to ensure that both the original and the translated version conveyed the same meaning. With the translated version, some adaptations were made to include all types of sexual orientations and gender identities, as the original questionnaire focused on LGB individuals (see Table 2). For example, the phrase “believed that homosexuality was immoral” was modified to “believed that being LGBTI+ was immoral.” Similarly, four items were removed because they focused solely on gay and lesbian individuals (“item 6–believed that marriage between homosexual individuals was unacceptable” and “item 11–would have had a problem seeing two homosexual people together in public”) or referred to behaviors that parents could do and not the other family members (“item 13–kicked me out of the house” and “item 28–severed financial support”). However, these eliminations have not affected the presence of the eight dimensions evaluated by the PPRS, as there are still items from all of them.

**TABLE 2:** Changes conducted in the adapted version.

Original version	Adapted version
Supported me	Supported me
2 Was worried about what his/her friends or other parents would think of him/her.	Was worried about what his/her friends or *acquaintances* would think of him/her.
3 Had the attitude that homosexual people should not work with children.	Had the attitude that *LGBTI+* people should not work with children.
4 Was concerned about what the family might think of him/her.	Was concerned about what the family might think of him/her.
5 Was proud of me.	Was proud of me.
6 Believed that marriage between homosexual individuals was unacceptable.	*Deleted*.
7 Was concerned about the potential that he/she would not get grandchildren from me.	Was concerned about *the fact that I would not be able to have children*.
8 Realized I was still “me,” even though I was gay/lesbian/bisexual.	Realized I was still “me,” even though I was *LGBTI+*.
9 Believed that homosexuality was immoral.	Believed that *being LGBTI+* was immoral.
10 Thought it was great.	Thought it was great.
11 Would have had a problem seeing two homosexual people together in public.	*Deleted*.
12 Was concerned about having to answer other people's questions about my sexuality.	Was concerned about having to answer other people's questions about my sexuality.
13 Kicked me out of the house.	*Deleted*.
14 Did not believe me.	Did not believe me.
15 Yelled and/or screamed.	Yelled and/or screamed.
16 Prayed to God, asking him to turn me straight.	Prayed to God, asking him to turn me *“normal.”*
17 Blamed himself/herself.	Blamed himself/herself.
18 Called me derogatory names, like “faggot” or “queer.”	Called me derogatory names, like “faggot”, *“dyke”, or “tranny.”*
19 Pretended that I was not gay/lesbian/bisexual.	Pretended that I was not *LGBTI+*.
20 Was angry at the fact I was gay/lesbian/bisexual.	Was angry at the fact I was *LGBTI+*.
21 Wanted me not to tell anyone else.	Wanted me not to tell anyone else.
22 Cried tears of sadness.	Cried tears of sadness.
23 Said I was no longer his/her child.	Said I was no longer his/her *family*.
24 Told me it was just a phase.	Told me it was just a phase.
25 Was mad at someone he/she thought had “turned me gay/lesbian/bisexual.”	Was mad at someone he/she thought had “turned me *LGBTI+*.”
26 Wanted me to see a psychologist who could “make me straight.”	Wanted me to see a psychologist who could “make me *normal*.”
27 Was afraid of being judged by relatives and friends.	Was afraid of being judged by relatives and friends.
28 Severed financial support.	*Deleted*.
29 Brought up evidence to show that I must not be gay/lesbian/bisexual, such as “You had a girlfriend/boyfriend; you can't be gay/lesbian/bisexual.”	Brought up evidence to show that I must not be *LGBTI+*, such as “You had a girlfriend/boyfriend; you can't be *LGBTI+*.”
30 Was mad at me for doing this to him/her.	Was mad at me for doing this to him/her.
31 Wanted me not to be gay/lesbian/bisexual.	Wanted me not to be *LGBTI+*.
32 Was ashamed of my homosexuality.	Was ashamed of my *LGBTI+ identity*.

*Note*: Changes in the adapted version are italicized.

Two recruitment methods were employed to obtain the final sample of 2627 individuals. First, between November 2019 and November 2020, various advertisements were disseminated on Facebook and Instagram in Spain, inviting people to participate in a study on sexuality. Upon clicking on the ads, participants were directed to an initial screen where they received information about the research's anonymous, voluntary, and confidential nature and were asked to provide informed consent. They were then able to proceed with answering the online Qualtrics questionnaire. This approach yielded a total of 2593 responses. Second, from December 2019 to April 2020, direct contact was made with Spanish LGBTI+ associations and organizations to introduce this research and request them to disseminate it through their networks. This strategy resulted in an additional 1900 responses. Thus, a total of 4493 responses were collected through this convenience sampling method. Out of those 4493 collected responses, 168 individuals were excluded due to not indicating their sexual orientation or gender identity. Additionally, the responses of 342 individuals (7.9% of total) who indicated not having come out to anyone were excluded, along with 145 (3.3% of total) who had not personally disclosed their LGBTI+ identity, 243 (5.6% of total) who reported that no one in their family knew about their identity, and another 129 (2.9% of total) who did not consider anyone in their family to be truly significant. Moreover, 642 individuals did not respond to some of the posed questions, so their responses were also eliminated. On the other hand, considering the inclusion criteria (being over 18 years old and residing in Spain), 51 responses from underage individuals and 146 responses from individuals outside of Spain were removed, resulting in 2627 valid responses. This research, registered under file number 12/2018, obtained approval from the Ethics Committee of the Universitat Jaume I (Castellón, Spain).

### Statistical analyses

Descriptive analyses were conducted using sociodemographic data. Additionally, chi‐square and *t* tests were performed to assess statistically significant differences between the EFA and CFA samples. For effect size, Cramer's *V* tests were calculated using SPSS statistical package (version 29.0), and Cohen's *d* was computed using G*Power software (Faul et al., 2007).

To examine the internal structure of the instrument, an exploratory factor analysis (EFA) was carried out using FACTOR 12.04.01 software (Ferrando & Lorenzo‐Seva, 2017). Subsequently, a confirmatory factor analysis (CFA) was conducted using the R software (R Core Team, 2023), specifically the lavaan R package (Rosseel, 2012), to assess the adequacy of the proposed factorial model, utilizing the Weighted Least Square Mean and Variance adjusted (WLSMV) estimator. Based on the recommendations of Izquierdo et al. (2014), EFA and CFA were performed on distinct samples. The samples for each analysis were randomly selected using the SPSS. Participants were assigned a decimal number between 0 and 100 through an option provided by the SPSS program. Those assigned numbers between 0 and 50 were included in the EFA sample, while those between 51 and 100 were included in the CFA sample. The characteristics of the two samples are presented in Table 1.

The assessment of the factorial model's goodness‐of‐fit involved various indices: Satorra–Bentler chi‐square (*χ* ^2^), general model significance (*p*), relative chi‐square (*χ* ^*2*^ */df*), root mean square error of approximation (RMSEA), comparative fit index (CFI), Tucker–Lewis index (TLI), and standardized root mean square residual (SRMR). Based on the criteria established by Bagozzi and Yi (2011), an excellent model fit was achieved when the *χ* ^*2*^ value was not significant (*p* > 0.05), *χ* ^*2*^ */df* was between 1 and 2, CFI and TLI were ≥0.95, and RMSEA and SRMR were ≤0.05. However, according to the less strict criteria proposed by Hooper et al. (2008), *χ* ^*2*^ */df* values between 2 and 3, CFI and TLI values ≥0.90, RMSEA values ≤0.08, and SRMR values ≤0.10 were also considered acceptable.

The analysis of structural invariance was conducted using the complete sample of 2627 individuals, utilizing the lavaan (Rosseel, 2012) and the semTools R package (Jorgensen et al., 2022). The aim was to investigate if there were any variations in the structure of the FRCO based on factors such as the participants' gender, the gender of the most significant relative, and the type of relative. For the participants' gender analysis, the comparison was between cisgender and transgender men versus cisgender and transgender women versus nonbinary individuals. The gender of the most relevant relative was compared between men (father, brother, uncle, grandfather, and male cousin) versus women (mother, sister, aunt, grandmother, and female cousin). In this case, 595 responses related to partners, children, and the “other” category were also eliminated, as their gender was unknown. Finally, a comparison was made based on the type of relative, comparing those who responded about their parents with those who responded about another family member (brother, sister, uncle, aunt, grandfather, grandmother, cousins, partner, children, or another family member). The fit of each of the invariance models was assessed by comparing pairs of nested models (△) using the RMSEA, CFI, and SRMR indices. A change ≥0.01 in CFI, ≥ 0.015 in RMSEA, or ≥0.03 in SRMR indicates a significant decrease in the model fit when testing for measurement invariance (Chen, 2007).

The internal consistency of the measure was evaluated using SPSS software (version 29.0), employing both Ordinal Cronbach's alpha (*α*) and McDonald's Omega (*ω*). For interpretation purposes, internal consistency values between 0.70 and 0.79 were deemed acceptable, between 0.80 and 0.89 were considered good, and values ≥0.90 were classified as excellent, following the guidelines Hunsley and Mash (2008) set.

Finally, to assess convergent validity, SPSS software (version 29.0) was used to calculate the correlations between FRCO and the three related single items (“fear of them getting angry,” “fear of being thrown out of the house,” and “fear of being punished in some way”).

## RESULTS

### Descriptive data

The mean score of the FRCO questionnaire was 46.36 (*SD* = 22.03), with a range of 28–136, with higher scores indicating a more negative reaction from the family member. On the other hand, as shown in Table 3, among the relatives to whom they had revealed their LGBTI+ identity, the mother was the most commonly chosen as the most relevant (43.1%), followed by the partner (20.6%) and the sister (13.9%).

**TABLE 3:** Frequency of the chosen most relevant family member.

	*n*	*%*
Mother	1133	43.1
Father	135	5.1
Sister	365	13.9
Brother	172	6.5
Partner	541	20.6
Children	43	1.6
Aunt	35	1.3
Uncle	3	0.1
Grandmother	65	2.5
Grandfather	5	0.2
Female cousin	96	3.7
Male cousin	23	0.9
Another family member	11	0.4

### Exploratory factor analysis

The Kaiser–Meyer–Olkin (KMO) index was >0.80 (KMO = 0.974), and Bartlett's test of sphericity (*χ* ^*2*^ = 15229.7, *df* = 378, *p* < 0.001) along with the determinant of the polychoric correlation matrix (<0.001) were statistically significant. These three indicators suggested that the structure of the data was adequate for factorial analysis.

Both the Parallel Analysis (Timmerman & Lorenzo‐Seva, 2011) and the Hull Method (Lorenzo‐Seva et al., 2011) suggested a unifactorial model. The results also suggested that the data could be treated as essentially unifactorial (Ferrando & Lorenzo‐Seva, 2018), supported by the fact that the Unidimensional Congruence exceeded 0.95 (UniCo = 0.990), the Explained Common Variance was higher than 0.85 (ECV = 0.931), and the Mean of Item Residual Absolute Loadings was below 0.30 (MIREAL = 0.188). Using the Robust Diagonally Weighted Least Squares (RDWLS) estimator, a good fit of the 1‐factor model was achieved (*χ* ^*2*^ = 2222.94; *df* = 350; *p* < 0.001; *χ* ^*2*^/*df* = 6.35; CFI = 0.994; TLI = 0.994; RMSEA = 0.063). It can be observed that the CFI and TLI values showed an excellent fit (>0.95), while the RMSEA value was deemed acceptable (<0.08). The unifactor model explained 68.72% of the total variance. Descriptive data of the FRCO items are presented in Table 4.

**TABLE 4:** Factor loadings and communalities for exploratory factor analysis of the FRCO.

	Factor loadings	Communality
Supported me	0.790	0.624
2 Was worried about what his/her friends or acquaintances would think of him/her.	0.716	0.513
3 Had the attitude that LGBTI+ people should not work with children.	0.738	0.544
4 Was concerned about what the family might think of him/her.	0.779	0.607
5 Was proud of me.	0.772	0.595
6 Was concerned about the fact that I would not be able to have children.	0.665	0.442
7 Realized I was still “me,” even though I was LGBTI+.	0.725	0.526
8 Believed that being LGBTI+ was immoral.	0.823	0.677
9 Thought it was great.	0.843	0.710
10 Was concerned about having to answer other people's questions about my sexuality.	0.740	0.548
11 Did not believe me.	0.707	0.500
12 Yelled and/or screamed.	0.870	0.756
13 Prayed to God, asking him to turn me “normal.”	0.852	0.726
14 Blamed himself/herself.	0.861	0.741
15 Called me derogatory names, like “faggot”, “dyke”, or “tranny.”	0.797	0.636
16 Pretended that I wasn't LGBTI+.	0.894	0.800
17 Was angry at the fact I was LGBTI+.	0.951	0.904
18 Wanted me not to tell anyone else.	0.873	0.762
19 Cried tears of sadness.	0.872	0.760
20 Said I was no longer his/her family.	0.728	0.530
21 Told me it was just a phase.	0.816	0.666
22 Was mad at someone he/she thought had “turned me LGBTI+.”	0.886	0.786
23 Wanted me to see a psychologist who could “make me normal.”	0.865	0.749
24 Was afraid of being judged by relatives and friends.	0.872	0.761
25 Brought up evidence to show that I must not be LGBTI+, such as “You had a girlfriend/boyfriend; you cannot be LGBTI+.”	0.797	0.635
26 Was mad at me for doing this to him/her.	0.933	0.871
27 Wanted me not to be LGBTI+.	0.944	0.892
28 Was ashamed of my LGBTI+ identity.	0.964	0.930
Variance	68.72	

### Confirmatory factor analysis

**FIGURE 1:**
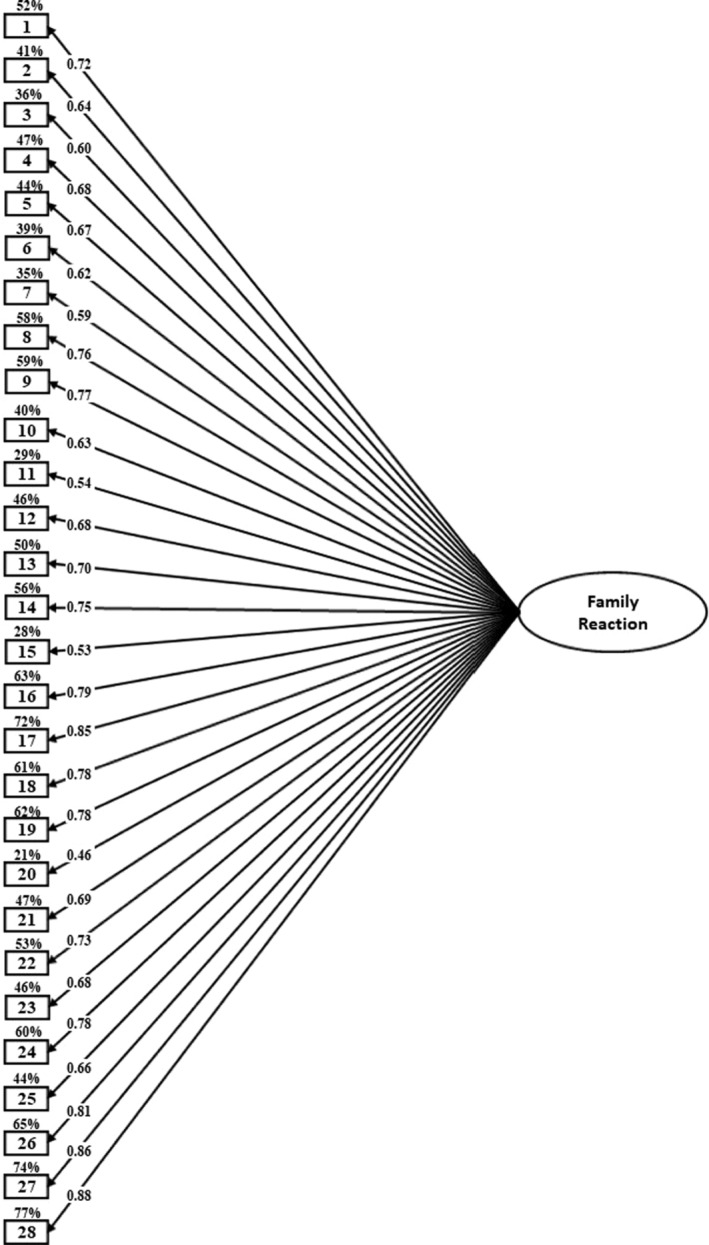
Confirmatory factor analysis for the family reaction to coming out (FRCO). *R* ^2^ is expressed as a percentage outside the main endogenous variables, represented by boxes. The coefficients are reported in standardized format. To enhance interpretation, error terms are not included.  *p* < 0.001.

A confirmatory factor analysis (CFA) was performed to verify the 1‐factor model's structure. The results obtained suggested a good fit of the model (*χ* ^*2*^ = 2500.89; *df* = 350; *p* < 0.001; *χ* ^*2*^/*df* = 7.15; RMSEA = 0.042; CFI = 0.984; TLI = 0.983; SRMR = 0.064). Specifically, the values of CFI, TLI, and RMSEA reached excellent levels (CFI and TLI >0.95, and RMSEA <0.05), and the SRMR was acceptable (SRMR <0.10). The resulting 1‐factor model is presented in Figure 1.

### Structural invariance

Initially, gender invariance of the chosen family member as the most relevant was tested. Table 5 displayed evidence supporting configural invariance, indicating equivalent model forms across groups (RMSEA = 0.044; CFI = 0.985; TLI = 0.984; SRMR = 0.062). Metric invariance, which examines the equivalence of factor loadings across groups, did not reveal any substantial deteriorations in the model fit for the chosen family member's gender (△RMSEA = 0.002; △CFI = 0.002; △SRMR = 0.007). Similarly, scalar invariance, which assesses the equivalence of item intercepts or thresholds across groups, did not show any significant decrease in the model fit (△RMSEA <0.001; △CFI <0.001; △SRMR <0.001). Lastly, during the examination of residual invariance (equivalence of items' residuals or unique variances across groups), there was no significant decrease in model fit observed (△RMSEA <0.001; △CFI <0.001; △SRMR = 0.002).

**TABLE 5:** Multigroup CFAs according to family member's gender (male vs female).

	*χ* ^2^	*df*	*p*	*χ* ^2^/*df*	RMSEA (90% CI)	CFI	TLI	SRMR	Comparisons	△ RMSEA	△ CFI	△ SRMR
Configural invariance	3645.20	700	<0.001	5.21	0.044 [0.043–0.046]	0.985	0.984	0.062	NA			
Metric invariance	1614.98	727	<0.001	2.22	0.046 [0.043–0.049]	0.983	0.983	0.069	M versus C	0.002	0.002	0.007
Scalar invariance	1639.66	754	<0.001	2.17	0.046 [0.043–0.049]	0.983	0.983	0.069	S versus M	<0.001	<0.001	<0.001
Residual invariance	1650.23	782	<0.001	2.11	0.045 [0.042–0.048]	0.983	0.983	0.071	R versus S	<0.001	<0.001	0.002

Abbreviations: CFA, confirmatory factor analysis; CFI, comparative fit index; *df*, degrees of freedom; *p*, general model significance; RMSEA, root mean square error of approximation; SRMR, standardized root mean square residual; TLI, Tucker–Lewis index; *χ* ^2^, Satorra–Bentler chi‐square; *χ* ^2^/*df*, normed chi‐square.

Additionally, we examined the invariance based on the participants' gender (male vs female vs nonbinary gender). In this case (see Table 6), we found that configural invariance was also supported, indicating equivalent model forms (RMSEA = 0.043; CFI = 0.984; TLI = 0.983; SRMR = 0.063). There were no significant deteriorations in the model fit concerning metric invariance (△RMSEA = 0.004; △CFI = 0.004; △SRMR = 0.011). Similarly, when assessing scalar invariance, there was no significant decrease in the model fit (△RMSEA = 0.001; △CFI = 0.002; △SRMR = 0.002). Finally, in residual invariance, the values indicate that the decrease in model fit was not significant (△RMSEA <0.001; △CFI = 0.002; △SRMR = 0.006).

**TABLE 6:** Multigroup CFAs according to the participants' gender (male vs female vs nonbinary gender).

	*χ* ^2^	*df*	*p*	*χ* ^2^/*df*	RMSEA (90% CI)	CFI	TLI	SRMR	Comparisons	△ RMSEA	△ CFI	△ SRMR
Configural invariance	3994.28	1050	<0.001	3.80	0.043 [0.041–0.044]	0.984	0.983	0.063	NA			
Metric invariance	2034.23	1104	<0.001	1.84	0.047 [0.004–0.050]	0.980	0.979	0.075	M versus C	0.004	0.004	0.011
Scalar invariance	2180.64	1158	<0.001	1.88	0.048 [0.045–0.052]	0.978	0.978	0.076	S versus M	0.001	0.002	0.002
Residual invariance	2268.13	1214	<0.001	1.86	0.049 [0.046–0.052]	0.976	0.978	0.083	R versus S	<0.001	0.002	0.006

Abbreviations: CFA, confirmatory factor analysis; CFI, comparative fit index; *df*, degrees of freedom; *p*, general model significance; RMSEA, root mean square error of approximation; SRMR, standardized root mean square residual; TLI, Tucker–Lewis index; *χ* ^2^, Satorra‐Bentler chi‐square; *χ* ^2^/*df*, normed chi‐square.

Table 7 presents the invariance comparing those who responded about a parent versus those who responded regarding another family member. The results also supported the configural invariance of this scale (RMSEA = 0.046; CFI = 0.982; TLI = 0.980; SRMR = 0.065). Metric invariance analysis revealed no significant deteriorations in the model fit concerning the type of family member (△RMSEA = 0.002; △CFI = 0.003; △SRMR = 0.007). When assessing scalar invariance, there was also no significant decrease in the model fit (△RMSEA = 0.001; △CFI = 0.002; △SRMR = 0.002). Finally, when testing residual invariance across type of family member, there was no significant decrease in model fit (△RMSEA = 0.002; △CFI = 0.003; △SRMR = 0.012).

**TABLE 7:** Multigroup CFAs according to type of family member (parents vs other family member).

	*χ* ^2^	*df*	*p*	*χ* ^2^/*df*	RMSEA (90% CI)	CFI	TLI	SRMR	Comparisons	△ RMSEA	△ CFI	△ SRMR
Configural invariance	4806.66	700	<0.001	6.87	0.044 [0.043–0.045]	0.984	0.982	0.063	NA			
Metric invariance	1797.15	727	<0.001	2.47	0.045 [0.042–0.047]	0.982	0.982	0.070	M versus C	0.001	0.001	0.007
Scalar invariance	1860.90	754	<0.001	2.47	0.045 [0.042–0.048]	0.981	0.981	0.071	S versus M	<0.001	0.001	0.001
Residual invariance	1882.43	782	<0.001	15.91	0.045 [0.042–0.048]	0.981	0.981	0.074	R versus S	<0.001	0.001	0.003

Abbreviations: CFA, confirmatory factor analysis; CFI, comparative fit index; *df*, degrees of freedom; *p*, general model significance; RMSEA, root mean square error of approximation; SRMR, standardized root mean square residual; TLI, Tucker–Lewis index; *χ* ^2^, Satorra–Bentler chi‐square; *χ* ^2^/*df*, normed chi‐square.

### Internal consistency

Both the values of Cronbach's alpha (*α*) and McDonald's Omega (ω) showed excellent internal consistency for the FRCO (*α* = 0.964; *ω* = 0.965). Moreover, the agreement between these two measures has been seen as a strong indicator of the scale's internal consistency in diverse circumstances (Zinbarg et al., 2005).

### Convergent validity

Out of the total of 2627 participants, 25.1% expressed fear that others would become angry upon revealing their sexual orientation, 10.6% feared being thrown out of their homes, and 9.7% feared being punished in some way. Supporting convergent validity, the FRCO showed positive correlations with fear of others getting angry (*r* = 0.187; *p* < 0.001), fear of being thrown out of the house (*r* = 0.189; *p* < 0.001), and fear of being punished in some way (*r* = 0.143; *p* < 0.001).

## DISCUSSION

The aim of this study was to adapt the PPRS (Willoughby et al., 2006, 2010) and create and validate a new version to assess the perceived reactions of any family member to coming out in Spain. Additionally, the intention was for the FRCO to be answerable by anyone belonging to a sexual or gender minority. Considering the obtained data, we could affirm that the FRCO is a reliable and valid measure with excellent psychometric properties.

In the PPRS (Willoughby et al., 2006, 2010), different theoretical domains were evaluated (negative shock, denial, anger, bargaining, depression, acceptance, general homophobia, and parent‐focused concerns). However, the authors proposed using the scale as a whole. The exploratory and confirmatory factor analyses conducted in this study support this, since a unifactorial model was obtained for the new created version.

Regarding internal consistency, the data obtained in the FRCO were similar to those obtained in the original version (Willoughby et al., 2006), as well as in other studies that used the PPRS (Bregman et al., 2013; Mitrani et al., 2017; Richter et al., 2017) or an adaptation of it (Li & Samp, 2021; Michli & Jamil, 2022).

Based on the results of invariance, the structure of the FRCO scale does not vary based on the gender of the respondent, the gender of the family member, or the type of family member (parent vs other family member). These results are very interesting because other studies have shown that others' reactions, beyond just parents, have an emotional impact on the individual coming out (Ryan et al., 2015). In fact, some researchers had already attempted to adapt the PPRS to assess the reactions of other family members, such as partners (Li & Samp, 2021).

The FRCO was related to having fear that others would become angry upon revealing their sexual orientation, feared being thrown out of their homes, and feared being punished in some way. In the Rejection Sensitivity Model, Feinstein (2020) posits that past experiences of discrimination can lead to expectations of future rejection and hypervigilance for signs of rejection. Additionally, individuals with rejection sensitivity would perceive rejection in the presence of minimal or ambiguous cues. Thus, people who fear familial rejection when coming out may have experienced discrimination from their families in the past, which would lead them to be more sensitive to any negative stimulus and to perceive ambiguous or minimally negative situations as rejection. This phenomenon would explain the higher FRCO scores among those who were afraid of coming out.

Despite the significant results, it is important to note some limitations of this study. First, this measure is based on the retrospective reports of the participants, which may introduce possible biases of memory. Moreover, it is important to note that perceived reactions were evaluated, not the events themselves. However, several studies have demonstrated the significant relationship between measures of perceived experiences, such as social support or discrimination, and mental health (Fan et al., 2021; Noret et al., 2020; Stickley et al., 2023). Second, due to the small sample size of gender minorities and the unbalanced samples, it was not methodologically appropriate to test invariance between sexual minorities and gender minorities, but for future studies, it would be convenient to analyze it. On the other hand, the reaction of friends to coming out has also been shown to be relevant for the emotional adjustment of those involved (Ryan et al., 2015; Shilo & Savaya, 2011), so it would be interesting to verify if this same questionnaire continues to demonstrate the same good psychometric properties when evaluating this population.

In conclusion, the results obtained in this study demonstrate that the FRCO scale could be an appropriate, valid, and useful tool for assessing the perception of the initial reactions that occurred when participants disclosed their sexual orientation or gender identity to their family members. This new version will allow for a comprehensive evaluation of perceived family reactions. As a result, the study of mental health issues stemming from perceived family rejection or the protective impact of family acceptance will be facilitated. Likewise, this questionnaire can help identify the typical reactions of family members, as well as those who tend to exhibit more negative responses. Furthermore, to the extent that it evaluates the perception that LGBTI+ people have of family reactions, and given that the perception of rejection situations modulates emotional reactions and mental health (Fan et al., 2021; Noret et al., 2020; Stickley et al., 2023), this questionnaire can help to understand the experience that LGBTI+ people have of this process and how this perception may have impacted their emotional well‐being. Such insights can be useful for health professionals engaged in family therapy and, in general, for any mental health professional who needs to work with the stressors experienced by their LGBTI+ patient or client. Furthermore, since this questionnaire can be answered by individuals of any sexual orientation or gender identity, comparative studies can be conducted to determine if there is any subgroup within the LGBTI+ community that experiences greater rejection, thus enabling intervention strategies to be targeted accordingly. Finally, this Spanish version that complements the original, which was written in English, will help it to be used also in Spanish‐speaking countries, reaching almost 600 million people.

## FUNDING INFORMATION

This research was supported by grant UJI‐B2018‐42 and PREDOC/2017/45 of the University Jaume I of Castellón (Spain).

## CONFLICT OF INTEREST STATEMENT

We have no conflict of interest to disclose.

## Supporting information


Appendix S1.

## References

[famp13047-bib-0001] Abou‐Chadi, T. , & Finnigan, R. (2019). Rights for same‐sex couples and public attitudes toward gays and lesbians in Europe. Comparative Political Studies, 52(6), 868–895. 10.1177/0010414018797947

[famp13047-bib-0002] Bagozzi, R. P. , & Yi, Y. (2011). Specification, evaluation, and interpretation of structural equation models. Journal of the Academy of Marketing Science, 40, 8–34. 10.1007/s11747-011-0278-x

[famp13047-bib-0003] Baiocco, R. , Fontanesi, L. , Santamaria, F. , Ioverno, S. , Baumgartner, E. , & Laghi, F. (2016). Coming out during adolescence: Perceived parents' reactions and internalized sexual stigma. Journal of Health Psychology, 21(8), 1809–1813. 10.1177/1359105314564019 25538141

[famp13047-bib-0004] Baiocco, R. , Fontanesi, L. , Santamaria, F. , Ioverno, S. , Marasco, B. , Baumgartner, E. , Willoughby, B. L. B. , & Laghi, F. (2015). Negative parental responses to coming out and family functioning in a sample of lesbian and gay young adults. Journal of Child and Family Studies, 24(5), 1490–1500. 10.1007/s10826-014-9954-z

[famp13047-bib-0005] Baiocco, R. , Marasco, B. , Astuto, A. , & Lonigro, A. (2012). Qualità della relazione con i genitori, funzionamento familiare e coming out in giovani gay e lesbiche [Parents' quality of relationship, family functioning and coming out in lesbian and gay young adults]. Counseling: Giornale Italiano di Ricerca e Applicazioni, 5(2), 193–206.

[famp13047-bib-0006] Bregman, H. R. , Malik, N. M. , Page, M. J. L. , Makynen, E. , & Lindahl, K. M. (2013). Identity profiles in lesbian, gay, and bisexual youth: The role of family influences. Journal of Youth and Adolescence, 42(3), 417–430. 10.1007/s10964-012-9798-z 22847752 PMC3500574

[famp13047-bib-0007] Chen, F. F. (2007). Sensitivity of goodness of fit indexes to lack of measurement invariance. Structural Equation Modeling: A Multidisciplinary Journal, 14(3), 464–504. 10.1080/10705510701301834

[famp13047-bib-0008] D'amico, E. , Julien, D. , Tremblay, N. , & Chartrand, E. (2015). Gay, lesbian, and bisexual youths coming out to their parents: Parental reactions and youths' outcomes. Journal of GLBT Family Studies, 11(5), 411–437. 10.1080/1550428X.2014.981627

[famp13047-bib-0009] D'Augelli, A. R. , Grossman, A. H. , & Starks, M. T. (2008). Families of gay, lesbian, and bisexual youth: What do parents and siblings know and how do they react? Journal of GLBT Family Studies, 4(1), 95–115. 10.1080/15504280802084506

[famp13047-bib-0010] European Union Agency for Fundamental Rights (2020). A long way to go for LGBTI equality. Publications Office of the European Union. https://fra.europa.eu/sites/default/files/fra_uploads/fra‐2020‐lgbti‐equality‐1_en.pdf

[famp13047-bib-0011] Fan, W. , Qian, Y. , & Jin, Y. (2021). Stigma, perceived discrimination, and mental health during China's COVID‐19 outbreak: A mixed‐methods investigation. Journal of Health and Social Behavior, 62(4), 562–581. 10.1177/00221465211040550 34605700 PMC8637388

[famp13047-bib-0012] Faul, F. , Erdfelder, E. , Lang, A. G. , & Buchner, A. (2007). G*power 3: A flexible statistical power analysis program for the social, behavioral, and biomedical sciences. Behavior Research Methods, 39(2), 175–191. 10.3758/BF03193146 17695343

[famp13047-bib-0013] Feinstein, B. A. (2020). The rejection sensitivity model as a framework for understanding sexual minority mental health. Archives of Sexual Behavior, 49(7), 2247–2258. 10.1007/s10508-019-1428-3 31286339 PMC8714401

[famp13047-bib-0014] Ferrando, P. J. , & Lorenzo‐Seva, U. (2017). Program FACTOR at 10: Origins, development and future directions. Psicothema, 29(2), 236–241. 10.7334/psicothema2016.304 28438248

[famp13047-bib-0015] Ferrando, P. J. , & Lorenzo‐Seva, U. (2018). Assessing the quality and appropriateness of factor solutions and factor score estimates in exploratory item factor analysis. Educational and Psychological Measurement, 78(5), 762–780. 10.1177/0013164417719308 32655169 PMC7328234

[famp13047-bib-0016] Gertler, L. M. (2014). The coming out experience, internalized homophobia and self‐compassion in LGBQ young adults [doctoral dissertation, the Wright Institute]. ProQuest Dissertations Publishing. https://www.proquest.com/dissertations‐theses/coming‐out‐experience‐internalized‐homophobia/docview/1790947566/se‐2

[famp13047-bib-0017] Habarth, J. M. (2008). Thinking “straight”: Heteronormativity and associated outcomes across sexual orientation [doctoral dissertation, University of Michigan]. ProQuest Dissertations Publishing. https://www.proquest.com/dissertations‐theses/thinking‐straight‐heteronormativity‐associated/docview/304571687/se‐2

[famp13047-bib-0018] Herz, M. , & Johansson, T. (2015). The normativity of the concept of heteronormativity. Journal of Homosexuality, 62(8), 1009–1020. 10.1080/00918369.2015.1021631 25710334

[famp13047-bib-0019] Holland, L. , Matthews, T. L. , & Schott, M. R. (2013). “That's so gay!” exploring college students' attitudes toward the LGBT population. Journal of Homosexuality, 60(4), 575–595. 10.1080/00918369.2013.760321 23469818

[famp13047-bib-0020] Hooper, D. , Coughlan, J. , & Mullen, M. R. (2008). Structural equation modeling: Guidelines for determining model fit. Electronic Journal of Business Research Methods, 6(1), 53–60.

[famp13047-bib-0021] Hunsley, J. , & Mash, E. J. (2008). A guide to assessments that work. Oxford University Press.

[famp13047-bib-0022] Izquierdo, I. , Olea, J. , & Abad, F. J. (2014). Exploratory factor analysis in validation studies: Uses and recommendations. Psicothema, 26(3), 395–400. 10.7334/psicothema2013.349 25069561

[famp13047-bib-0023] Jorgensen, T. D. , Pornprasertmanit, S. , Schoemann, A. M. , & Rosseel, Y. (2022). semTools: Useful tools for structural equation modeling. R Package Version 0.5–6. https://CRAN.R‐project.org/package=semTools

[famp13047-bib-0024] Li, Y. , & Samp, J. A. (2021). Predictors and outcomes of LGB individuals' sexual orientation disclosure to heterosexual romantic partners. Journal of Applied Communication Research, 49(1), 24–43. 10.1080/00909882.2020.1849769

[famp13047-bib-0025] Lorenzo‐Seva, U. , Timmerman, M. E. , & Kiers, H. A. L. (2011). The Hull method for selecting the number of common factors. Multivariate Behavioral Research, 46(2), 340–364. 10.1080/00273171.2011.564527 26741331

[famp13047-bib-0026] Maneesriwongul, W. , & Dixon, J. K. (2004). Instrument translation process: A methods review. Journal of Advanced Nursing, 48(2), 175–186. 10.1111/j.1365-2648.2004.03185.x 15369498

[famp13047-bib-0027] Matthews, C. H. , & Salazar, C. F. (2012). An integrative, empowerment model for helping lesbian, gay, and bisexual youth negotiate the coming‐out process. Journal of LGBT Issues in Counseling, 6(2), 96–117. 10.1080/15538605.2012.678176

[famp13047-bib-0028] McCurdy, A. L. , Lavner, J. A. , & Russell, S. T. (2023). A latent profile analysis of perceived family reactions to youth LGBTQ identity. Journal of Family Psychology, 37(6), 888–898. 10.1037/fam0001114 37199940 PMC10524290

[famp13047-bib-0029] Michli, S. , & Jamil, F. E. (2022). Internalized homonegativity and the challenges of having same‐sex desires in the Lebanese context: A study examining risk and protective factors. Journal of Homosexuality, 69(1), 75–100. 10.1080/00918369.2020.1809893 32910742

[famp13047-bib-0030] Mitrani, V. B. , De Santis, J. P. , McCabe, B. E. , Deleon, D. A. , Gattamorta, K. A. , & Leblanc, N. M. (2017). The impact of parental reaction to sexual orientation on depressive symptoms and sexual risk behavior among Hispanic men who have sex with men. Archives of Psychiatric Nursing, 31(4), 352–358. 10.1016/j.apnu.2017.04.004 28693870 PMC5721521

[famp13047-bib-0031] Noret, N. , Hunter, S. C. , & Rasmussen, S. (2020). The role of perceived social support in the relationship between being bullied and mental health difficulties in adolescents. School Mental Health: A Multidisciplinary Research and Practice Journal, 12(1), 156–168. 10.1007/s12310-019-09339-9

[famp13047-bib-0032] Pew Research Center (2020). The global divide on homosexuality persists. https://www.pewresearch.org/global/2020/06/25/global‐divide‐on‐homosexuality‐persists/

[famp13047-bib-0033] Puckett, J. A. , Woodward, E. N. , Mereish, E. H. , & Pantalone, D. W. (2015). Parental rejection following sexual orientation disclosure: Impact on internalized homophobia, social support, and mental health. LGBT Health, 2(3), 265–269. 10.1089/lgbt.2013.0024 26788675

[famp13047-bib-0034] R Core Team . (2023). R: A language and environment for statistical computing. R Foundation for Statistical Computing. https://www.R‐project.org/

[famp13047-bib-0035] Rama, J. , Zanotti, L. , Turnbull‐Dugarte, S. J. , & Santana, A. (2021). VOX: The rise of the Spanish populist radical right. Routledge. 10.4324/9781003049227

[famp13047-bib-0036] Richter, B. E. J. , Lindahl, K. M. , & Malik, N. M. (2017). Examining ethnic differences in parental rejection of LGB youth sexual identity. Journal of Family Psychology, 31(2), 244–249. 10.1037/fam0000235 27571323 PMC5328793

[famp13047-bib-0037] Rodríguez‐Temiño, I. , & Almansa‐Sánchez, J. (2021). The use of past events as political symbols in Spain. The example of Vox and the need for a new archaeology of ethnicity. International Journal of Heritage Studies, 27(10), 1064–1078. 10.1080/13527258.2021.1941195

[famp13047-bib-0038] Rosseel, Y. (2012). Lavaan: An R package for structural equation modeling. Journal of Statistical Software, 48(2), 1–36. 10.18637/jss.v048.i02

[famp13047-bib-0039] Ryan, W. S. , Legate, N. , & Weinstein, N. (2015). Coming out as lesbian, gay, or bisexual: The lasting impact of initial disclosure experiences. Self and Identity, 14(5), 549–569. 10.1080/15298868.2015.1029516

[famp13047-bib-0040] Schilt, K. , & Westbrook, L. (2009). Doing gender, doing heteronormativity: “Gender normals,” transgender people, and the social maintenance of heterosexuality. Gender & Society, 23(4), 440–464. 10.1177/0891243209340034

[famp13047-bib-0041] Shilo, G. , & Savaya, R. (2011). Effects of family and friend support on LGB youths' mental health and sexual orientation milestones. Family Relations, 60(3), 318–330. 10.1111/j.1741-3729.2011.00648.x

[famp13047-bib-0042] Stickley, A. , Shirama, A. , & Sumiyoshi, T. (2023). Perceived discrimination and mental health in the Japanese general population. International Journal of Social Psychiatry, 69(7), 1790–1800. 10.1177/00207640231175248 37300412

[famp13047-bib-0043] Timmerman, M. E. , & Lorenzo‐Seva, U. (2011). Dimensionality assessment of ordered polytomous items with parallel analysis. Psychological Methods, 16(2), 209–220. 10.1037/a0023353 21500916

[famp13047-bib-0044] Walker, M. D. (2016). How black LGBQ youths' perceptions of parental acceptance and rejection are associated with their self‐esteem and mental health [doctoral dissertation, Drexel University]. ProQuest Information & Learning. https://www.proquest.com/dissertations‐theses/how‐black‐lgbq‐youths‐perceptions‐parental/docview/1708665312/se‐2

[famp13047-bib-0045] Wigderson, S. , Lindahl, K. M. , & Malik, N. M. (2019). Parental responsiveness toward GLB children: Impact on mental health two years later. Journal of GLBT Family Studies, 15(4), 326–341. 10.1080/1550428X.2018.1545620 33033471 PMC7540723

[famp13047-bib-0046] Willoughby, B. L. , Doty, N. D. , Braaten, E. B. , & Malik, N. M. (2010). Perceived parental reactions scale. In T. D. Fisher , C. M. Davis , W. L. Yarber , & S. L. Davis (Eds.), Handbook of sexuality‐related measures (pp. 432–434). Routledge.

[famp13047-bib-0047] Willoughby, B. L. B. , Malik, N. M. , & Lindahl, K. M. (2006). Parental reactions to their sons' sexual orientation disclosures: The roles of family cohesion, adaptability, and parenting style. Psychology of Men & Masculinity, 7(1), 14–26. 10.1037/1524-9220.7.1.14

[famp13047-bib-0048] Worthen, M. G. (2012). Understanding college student attitudes toward LGBT individuals. Sociological Focus, 45(4), 285–305. 10.1080/00380237.2012.712857

[famp13047-bib-0049] Zinbarg, R. E. , Revelle, W. , Yovel, I. , & Li, W. (2005). Cronbach's *α*, Revelle's *β*, and Mcdonald's *ω* H: Their relations with each other and two alternative conceptualizations of reliability. Psychometrika, 70(1), 123–133. 10.1007/s11336-003-0974-7

