# Supplementary material for: Family reaction to coming out (FRCO): A Spanish adaptation and validation of perceived parental reactions scale
Source: Fam Process. 2024 Aug 8;64(1):e13047. doi: 10.1111/famp.13047 (PMC11781997; doi:10.1111/famp.13047)
Supplement: Supplementary file 1 — Appendix S1. [file FAMP-64-0-s001.docx]

**Supplemental Material**

Family Reaction to Coming Out (FRCO) – English version

(Willoughby, Malik, & Lindahl, 2006; adapted by Nebot-Garcia et al., 2024)

**INSTRUCTIONS:** Think back to the week when that person first became aware of your sexual orientation or gender identity. Read the following statements and indicate how much you agree or disagree with each statement. Remember, there are no correct or incorrect answers. These are your opinions.

| Strongly Disagree | Disagree | Neutral | Agree | Strongly Agree |
| --- | --- | --- | --- | --- |
| **1** | **2** | **3** | **4** | **5** |

**The week when that person found out that I was LGBTI+, he/she:**

1. Supported me.
2. Was worried about what his/her friends or acquaintances would think of him/her.
3. Had the attitude that LGBTI+ people should not work with children.
4. Was concerned about what the family might think of him/her. 5. I w
5. Was proud of myself.
6. Was concerned about the fact that I wouldn’t be able to have children.
7. Realized I was still "me," even though I was LGBTI+.
8. Believed that being LGBTI+ was immoral.
9. Thought it was great.
10. Was concerned about having to answer other people's questions about my sexuality.
11. Didn’t believe me.
12. Yelled and/or screamed.
13. Prayed to God, asking him to turn me "normal."
14. Blamed himself/herself.
15. Called me derogatory names, like “faggot”, “dyke”, or “tranny.”
16. Pretended that I wasn’t LGBTI+.
17. Was angry at the fact I was LGBTI+.
18. Wanted me not to tell anyone else.
19. Cried tears of sadness.
20. Said I was no longer his/her family.
21. Told me it was just a phase.
22. Was mad at someone he/she thought had "turned me LGBTI+."
23. Wanted me to see a psychologist who could "make me normal."
24. Was afraid of being judged by relatives and friends.
25. Brought up evidence to show that I must not be LGBTI+, such as "You had a girlfriend/boyfriend; you can’t be LGBTI+."
26. Was mad at me for doing this to him/her.
27. Wanted me not to be LGBTI+.
28. Was ashamed of my LGBTI+ identity.

Family Reaction to Coming Out (FRCO) – Spanish version

(Willoughby, Malik, & Lindahl, 2006; adapted by Nebot-Garcia et al., 2024)

**INSTRUCCIONES:** Piensa en la semana en que esa persona supo por primera vez sobre tu orientación sexual o identidad de género. Lee las siguientes declaraciones e indica en qué medida estás de acuerdo o en desacuerdo. Recuerda, no hay respuestas correctas o incorrectas. Estas son tus opiniones.

| Muy en desacuerdo | En desacuerdo | Neutral | De acuerdo | Muy de acuerdo |
| --- | --- | --- | --- | --- |
| **1** | **2** | **3** | **4** | **5** |

**La semana en que esa persona supo que yo era LGBTI+, él/ella:**

1. Me apoyó.
2. Estaba preocupado/a por lo que sus amistades o conocidos/as pensarían de él/ella.
3. Tenía la actitud de que las personas LGBTI+ no deberían trabajar con niños.
4. Estaba preocupado/a por lo que la familia podía pensar de él/ella.
5. Estaba orgulloso/a de mí.
6. Estaba preocupado/a sobre el hecho de que yo no podría tener descendencia.
7. Se dio cuenta de que seguía siendo “yo”, incluso siendo LGBTI+.
8. Creía que ser LGBTI+ era inmoral.
9. Pensó que estaba bien.
10. Estaba preocupado/a por tener que responder preguntas de otras personas sobre mi sexualidad.
11. No me creyó.
12. Me gritó.
13. Rezó a Dios, pidiéndole que me volviera “normal”.
14. Se culpó a él/ella mismo/a.
15. Me llamó de formas denigrantes, como “maricón”, “bollera” o “travelo”.
16. Pretendió que yo no fuera LGBTI+.
17. Estaba enfadado/a por el hecho de que fuera LGBTI+.
18. Quería que no se lo contase a nadie más.
19. Lloró de tristeza.
20. Dijo que ya no iba a seguir siendo su familia.
21. Me dijo que eso solo era una fase.
22. Estaba enfadado/a con la persona que él/ella creía que “me había vuelto LGBTI+”.
23. Quería que yo fuera a un psicólogo para que “me volviera normal”.
24. Tenía miedo de ser juzgado/a por sus parientes y amistades.
25. Me dio evidencias para demostrarme que yo no podía ser LGBTI+, tales como “tuviste un novio/a, no puedes ser LGBTI+”
26. Estaba enfadado/a conmigo por hacerle esto a él/ella.
27. Quería que yo no fuera LGBTI+.
28. Estaba avergonzado/a de mi identidad LGBTI+.
